# Ultrathin Polyimide Membrane as Cell Carrier for Subretinal Transplantation of Human Embryonic Stem Cell Derived Retinal Pigment Epithelium

**DOI:** 10.1371/journal.pone.0143669

**Published:** 2015-11-25

**Authors:** Tanja Ilmarinen, Hanna Hiidenmaa, Peeter Kööbi, Soile Nymark, Anni Sorkio, Jing-Huan Wang, Boris V. Stanzel, Fabian Thieltges, Päivi Alajuuma, Olli Oksala, Marko Kataja, Hannu Uusitalo, Heli Skottman

**Affiliations:** 1 BioMediTech, University of Tampere, Tampere, Finland; 2 Department of Ophthalmology, SILK, University of Tampere and Tays Eye Center, Tampere, Finland; 3 Department of Electronics and Communications Engineering and BioMediTech, Tampere University of Technology, Tampere, Finland; 4 Department of Ophthalmology, University of Bonn, Bonn, Germany; 5 Santen Oy, Tampere, Finland; 6 Tays Eye Center, Tampere, Finland; University of Newcastle upon Tyne, UNITED KINGDOM

## Abstract

In this study, we investigated the suitability of ultrathin and porous polyimide (PI) membrane as a carrier for subretinal transplantation of human embryonic stem cell (hESC) -derived retinal pigment epithelial (RPE) cells in rabbits. The *in vivo* effects of hESC-RPE cells were analyzed by subretinal suspension injection into Royal College of Surgeons (RCS) rats. Rat eyes were analyzed with electroretinography (ERG) and histology. After analyzing the surface and permeability properties of PI, subretinal PI membrane transplantations with and without hESC-RPE were performed in rabbits. The rabbits were followed for three months and eyes analyzed with fundus photography, ERG, optical coherence tomography (OCT), and histology. Animals were immunosuppressed with cyclosporine the entire follow-up time. In dystrophic RCS rats, ERG and outer nuclear layer (ONL) thickness showed some rescue after hESC-RPE injection. Cells positive for human antigen were found in clusters under the retina 41 days post-injection but not anymore after 105 days. In rabbits, OCT showed good placement of the PI. However, there was loss of pigmentation on the hESC-RPE-PI over time. In the eyes with PI alone, no obvious signs of inflammation or retinal atrophy were observed. In the presence of hESC-RPE, mononuclear cell infiltration and retinal atrophy were observed around the membranes. The porous ultrathin PI membrane was well-tolerated in the subretinal space and is a promising scaffold for RPE transplantation. However, the rejection of the transplanted cells seems to be a major problem and the given immunosuppression was insufficient for reduction of xenograft induced inflammation.

## Introduction

Retinal pigment epithelium (RPE) is a monolayer of cells between the neural retina and the choriocapillaris. It is vital as part of the blood-retina-barrier. It also supports photoreceptor function and survival by providing nutrients, absorbing stray light, phagocytosing photoreceptor outer segments, and controlling regeneration of visual pigments, ion flow, and oxidative stress [1].

RPE degeneration has a major role in pathogenesis of retinal diseases including age-related macular degeneration (AMD), a leading cause of blindness in developed societies [2]. In AMD, local degeneration of RPE eventually leads to death of photoreceptors [3]. A promising future treatment for AMD is cell therapy and submacular transplantation of RPE, which has been studied extensively [4]. Human pluripotent stem cells (hPSCs) are potential and readily available source for RPE replacement [5, 6]. Recent stem cell -based clinical trials for RPE-related diseases aim to establish safety and dosing with RPE cell suspensions derived from human embryonic stem cells (hESCs) [7, 8]. However, concerns remain that suspended RPE may fail to survive or function in the long-term on the diseased Bruch’s membrane [9, 10]. Another approach under clinical trial in Japan is transplantation of autologous human induced pluripotent stem cell (hiPSC) -derived RPE sheets without a supporting artificial scaffold [11, 12].

Biomaterial based carriers could facilitate surgical handling of intact RPE and ensure better long-term function of the transplanted cells [11–13]. Several materials have been proposed for RPE transplantation [14–17]. However, to date, transplantations of human RPE-scaffolds have been reported only with adult and fetal RPE cells on polyester in rabbits [18, 19] and with hPSC-RPE cells on parylene-C in rats [13, 20]. Despite encouraging results, further studies are needed to improve the biocompatibility of the scaffolds. One potential material is synthetic polymer polyimide (PI). Depending on its structure, PI has been clinically approved and its ocular biocompatibility has been demonstrated [21, 22]. Previously, PI membranes have been tested for subretinal transplantation in rats and pigs [23, 24]. We have also demonstrated that PI supports culture of hESC-RPE [25].

In this study, we aimed to further characterize the suitability of ultrathin and porous PI for subretinal transplantation of hESC-RPE. First, we assessed the *in vivo* effects of the hESC-RPE cells by injecting them subretinally in Royal College of Surgeons (RCS) rats, an FDA approved animal model for retinal degeneration [26]. For PI membrane transplantations, a larger eyed animal model rabbit was chosen for evaluation of surgical feasibility of the membrane. To our knowledge, this is the first report of *in vivo* studies with hPSC-RPE-biomaterial sheet transplantation in larger animals.

## Materials and Methods

### Surface and permeability characterization of ultrathin PI membrane

The properties of ultrathin (7.6 μm) PI membranes (pore diameter 1 μm, pore density 2.2 x 10^7^ pores/cm^2^; it4ip, Seneffe, Belgium) were examined with scanning electron microscopy (SEM), atomic force microscopy (AFM), contact angle analysis, and Ussing chamber system. A commonly used RPE culture substrate, polyethylene terephthalate (PET) membrane, was analyzed for comparison.

The pore distributions were determined by SEM (Quanta 3D, FEI, UK) operating at 5 kV. Samples were sputter coated with a thin layer of gold, using an Emitech K500X (Quorum Technologies, UK) to reduce charging and image distortion.

AFM (Nanoscope Dimension 3100, Vecco, USA) was equipped with a TESPA silicon tip (Vecco) mounted on a cantilever of stiffness of 20–80 N/m^-1^, operating at a resonance of 300 Hz and a scan rate of 0.996 Hz. Images were acquired in tapping mode. Root mean square average (Rq) values were calculated from 12 scan areas of 2 μm x 2 μm. Images were analyzed with the Nanoscope 6.11r1 Software (Vecco).

Static contact angle (CAM2000, KSV Instrument Ltd., Finland) measurements were taken using a 5 μl drop of Milli-Q water. Ten to 13 readings were performed per membrane type.

Flux of a small molecular weight (700 Da) Alexa Fluor® 568 Hydrazide sodium salt (Life Technologies, Paisley, UK) at a concentration of 0.0065 mM was measured in Ussing chamber system (Physiologic Instruments, San Diego, CA) as described previously [16]. Samples were collected from the receptor chamber at 60, 120, 180 and 240 min. The diffusion of Alexa Fluor® 568 Hydrazide sodium salt across the membranes was characterized by calculating the apparent permeability coefficient (Papp, cm^2^s^-1^) as Papp = dC/dt/(60C0A), where dC/dt is the slope of the linear portion of the permeability curve, C0 is the initial concentration in the donor chamber, and A is the exposed surface area of the hESC-RPE monolayer (0.031 cm^2^). The cumulative permeability demonstrates the percentage of diffused fluorescent marker in the receptor chamber compared to initial concentration in the donor chamber over time.

### Culture of hESC lines and RPE differentiation

The National Authority for Medicolegal Affairs Finland has approved our research with human embryos (Dnro 1426/32/300/05). We also have a supportive statement from the local ethics committee of the Pirkanmaa hospital district Finland to derive and expand hESC lines for research purposes (R05116).

Human ESC lines Regea08/023 and Regea08/017, which were previously derived in our laboratory [27], were cultured as previously described [28]. The hESC-RPE differentiation was performed spontaneously in floating cell clusters using RPEbasic method as described previously [28]. For enrichment, the pigmented areas were isolated manually using a scalpel. Subsequently, cells were dissociated with 1x Trypsin-EDTA (Lonza, Basel, Switzerland) or Tryple Select (Life Technologies), filtered through 40 μm cell strainer (BD Biosciences, NJ, USA), and seeded 160 000–200 000 cells/cm^2^ on collagen IV -coated (human placenta, 5 μg/cm^2^; Sigma-Aldrich, MO, USA) 24-well plates (NUNC, Thermo Fisher Scientific, Tokyo, Japan). After enrichment, the pigmented cells were replated on collagen IV -coated (5 μg/cm^2^) 24-well plates from which the cells were dissociated for injections. For PI membrane transplantation, the cells were replated on laminin-coated (human placenta, 10 μg/cm^2^; Sigma-Aldrich) PI.

### Transplantation studies

All animal experiments were approved by the Finnish National Animal Experiment Board (STH832A and PH398A) and the state regulatory authorities of North Rhine-Westphalia (LANUV 84–02.04.2014.A082), and were performed in accordance with the ARVO Statement for the Use of Animals in Ophthalmic and Vision research. All efforts were made to minimize suffering. The animals were maintained in temperature controlled environment in a 12 h light-dark cycle with free access to water.

### Cell suspension injection

These protocols have been described in detail in S1 Materials and Methods.

Prior injection, the expression of RPE/eye-related genes and proteins were analyzed from the hESC-RPE cells with reverse transcription polymerase chain reaction (RT-PCR) and immunocytochemistry. Passage three hESC-RPE cells (100 000 cells per injection) were used for subretinal injections into dystrophic and non-dystrophic RCS rats. The study groups were 1) no injection (n = 1 eye/rat strain), 2) injection of RPEbasic medium without cells (n = 1 eye/rat strain), 3) injection of hESC-RPE in RPEbasic medium (n = 4 eyes/dystrophic and n = 3 eyes/non-dystrophic). The viability of cells left over from injections and kept in suspension for 6 h at +4°C was analyzed with LIVE/DEAD® Cell Viability Assay kit (Life Technologies).

### PI membrane transplantation

For PI membrane transplantation, 1 × 4 mm bullet shaped implants were manually cut with a scalpel from laminin-coated PI membranes without and with hESC-RPE. The membranes were kept at +37°C until transplanted.

Nine albino New Zealand White rabbits weighing 3.1–3.9 kg were used. Due to the long follow-up time, systemic administration of immunosuppressant was chosen instead of repeated intraocular injections. The rabbits received 25 mg/kg cyclosporin A (Novartis, Basel, Switzerland) in drinking water starting two days before the transplantation until the end of follow-up (three months). The volume of water consumed by the animals was recorded daily. The rabbits were anesthetized with an intramuscular injection of 5 mg/kg xylazine (Bayer AG, Leverkusen, Germany) and 25 mg/kg ketamine (Parke-Davis Scandinavia AB, Solna, Sweden). Anesthesia was maintained with intravenously administered xylazine/ketamine. Topical anesthesia was performed with oxybuprocaine hydrochloride (Santen, Osaka, Japan) and the pupils were dilated with tropicamid (Santen) and 2.5% phenylephrine hydrochloride (Bausch & Lomb, NJ, USA).

Limbal conjunctival openings and 20 gauge sclerotomies were made into inferotemporal, inferonasal and superior segments of the right eye for infusion cannula, endoillumination, and other surgical instrumentation, respectively. Partial vitrectomy was performed using Accurus vitrectomy system (Alcon Laboratories, TX, USA). A small posterior retinotomy was made using 27 gauge injection needle tip and a small retinal bleb was gently raised by injecting balanced salt solution (BSS) into subretinal space. PI membrane alone (n = 3) or with hESC-RPE cells (hESC-RPE-PI, n = 5) was placed into subretinal space via superior sclerotomy and retinotomy using 23 gauge Grieshaber forceps (Alcon Labs, TX, USA). Retinal bleb was then reattached by passive aspiration of subretinal fluid via silicone tip 20 gauge cannula and vitreous cavity filled with 5000-centistoke silicone oil. Sclerotomies and conjunctival openings were closed using 7–0 Vicryl sutures. One animals underwent surgery without delivery of PI membrane (surgical control).

In order to better protect the hESC-RPE on PI and aid surgical manageability of the membrane, we evaluated the suitability of a custom-made shooter instrument [19] for delivery of ultrathin PI into the subretinal space of rabbits (n = 6, pigmented chinchilla bastard, >1.5kg), according to previously published surgical techniques [18]. In brief, following a 2 port 23G vitrectomy with the Mach 2 cutter attached to a Megatron S4 MPS device and 25G chandelier illumination connected to a Xenotron 3 light machine (all Geuder SG, Heidelberg, Germany), which included manual induction of posterior vitreous detachment, the surgeon (BVS) attempted to pass implants without cells through an enlarged retinotomy in a bleb retinal detachment into the subretinal space.

### Post-transplantation analyses

#### Electroretinography (ERG) measurements and optical coherence tomography (OCT) imaging

Dark-adapted Ganzfeld ERGs were recorded under dim red light from the rats (one to two animals per study group) 37 days post-injection and from all the rabbits before surgery and 15–17, 35–39, and 73–79 days after transplantation. Systemic and topical anesthesia and dilation of the pupils were performed as mentioned above (rabbits) or in S1 Materials and Methods (rats). ERG recordings were performed using a portable handheld multi-species Electroretinograph Model 2000 Unit (HMsERG, OcuScience, NV, USA). The eyes were stimulated with a mini-Ganzfeld dome either bilaterally with both eyes inside the dome (rats) or unilaterally with the center of the dome positioned 2 cm from the tested eye (rabbits). As a recording electrode, an ERG-jet contact lens electrode with gold mylar film was used for rabbits and a silver-embedded thread electrode with mini contact lens for rats (both from OcuScience). The ground and reference electrodes were stainless steel needle electrodes.

The recordings were performed in a Faraday cage. In rabbits, the ground electrode was placed subcutaneously over the external occipital protuberance and the reference electrodes were placed a few centimeters caudal to the lateral canthus. In rats, the ground electrode was placed subcutaneously above the base of the tail and the reference electrodes were placed subcutaneously in each cheek. The recording electrodes were placed on the cornea with 2% methylcellulose. Scotopic flash ERGs were recorded at 1, 3, 10, 30, 100, 300, 1000, 3000, 10000, and 25000 mcds/m^2^. Signals were amplified, digitized, averaged, and stored using ERGView 4.380R software (OcuScience). The analysis was performed with ERGView 4.380R software by evaluating the ERG waveforms as well as by measuring the amplitudes and implicit times for the a- and b-waves.

Three months after transplantation, OCT was performed to rabbits using Stratus OCT scanner, (Carl Zeiss Meditec, Dublin, CA, USA).

#### Retinal histology and immunohistochemistry

The animals were humanely euthanized at the end of the experiment with carbon dioxide (rats) or with overdose injection of pentobarbital sodium (rabbits). The eyes were fixed in Davidson’s fixative and processed for paraffin embedding following standard techniques and cut into five-micron sections. For histology, specimens were conventionally stained with haematoxylin and eosin, and observed under a microscope (Nikon Instruments Europe, Amsterdam, Netherlands). ONL thickness was measured from three separate sections per eye with ImageJ Processing and Analysis Software [29]. A total of 10–12 measurements were taken per section. From manipulated eyes, 5–6 measurements were taken from the graft site and 5–6 distally from the graft site with 100–200 μm intervals.

For immunohistochemistry, the samples were deparaffinated, hydrated and stained using conventional protocols. Antigen retrieval was performed by boiling slides for 5 min in 0.01 M citrate buffer (pH 6.0). Primary antibody information is listed in S1 Table. Primary antibodies were diluted 1:50 (anti-CRALBP, anti-CD68, anti-CD3, anti-TRA-1-85) for overnight at +4°C. Alexa Fluor 568-conjugated donkey anti-mouse IgG and Alexa Fluor 488-conjugated donkey anti-goat IgG (both 1:400, Life Technologies) were used as secondary antibodies. Sections not incubated with primary antibodies served as negative controls. Images were taken using Olympus IX51 fluorescence microscope (Olympus, Tokyo, Japan).

#### Statistical analysis

Statistical analysis between two groups was performed with the unpaired Mann-Whitney U test using IBM SPSS Statistics software. A p value of < 0.05 was considered statistically significant.

## Results

### Suspension injection of hESC-RPE into rat eye

Prior injection, hESC-RPE cells expressed several RPE markers both at the RNA (S1A Fig) and protein (S1B Fig) level and did not show expression of pluripotency markers *OCT4* and *NANOG* RNA (S1A Fig). Majority of the cells were still alive in suspension at the time of injection based on live/dead staining (S1C Fig).

**Fig 1:**
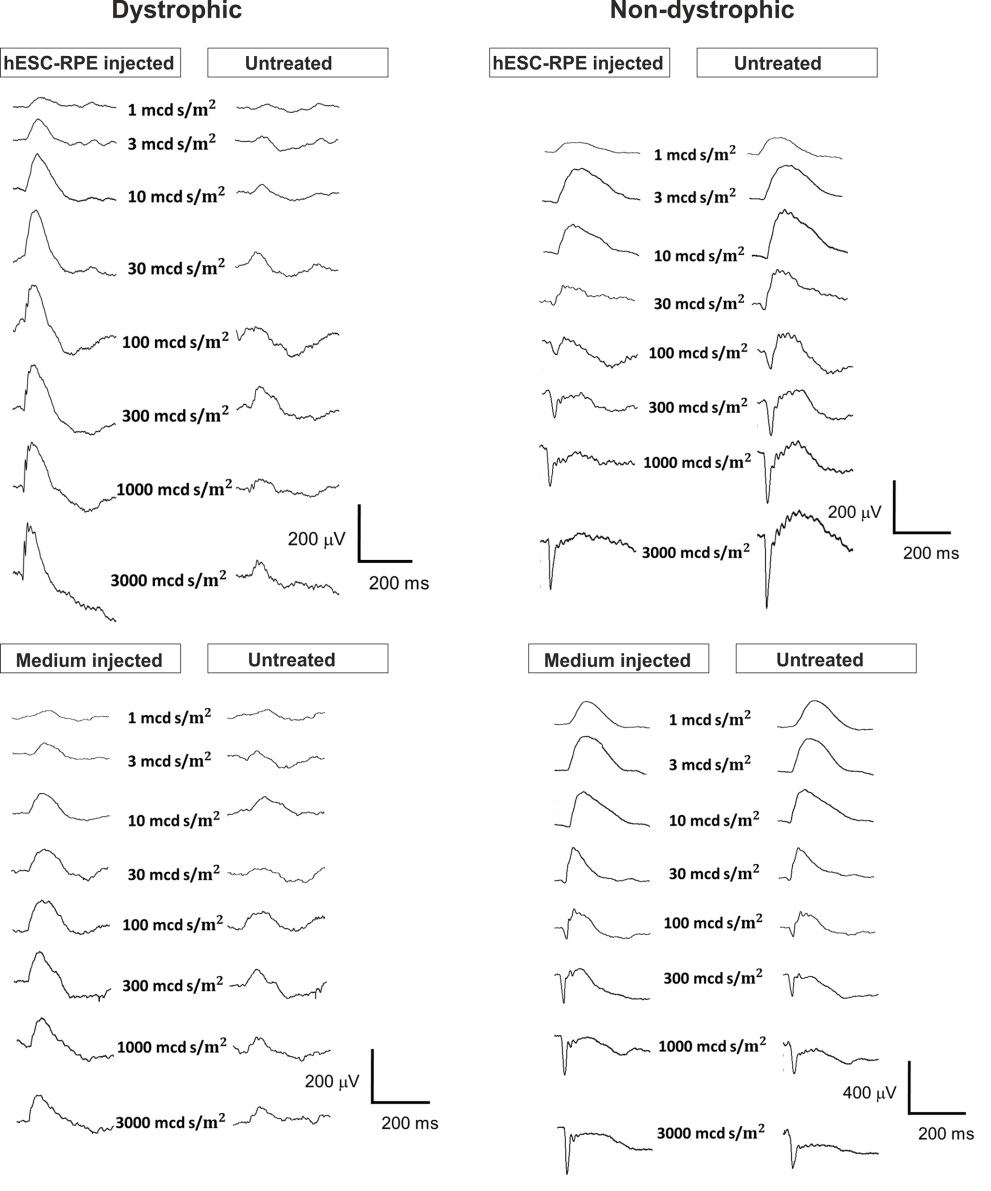
Dark-adapted ERG responses following subretinal injections in dystrophic and non-dystrophic RCS rats. Representative and averaged (2 to 20 responses) ERG recordings from dystrophic and non-dystrophic rats 37 days after subretinal injection of hESC-RPE or medium alone compared to the non-operated fellow eye.

**Fig 2:**
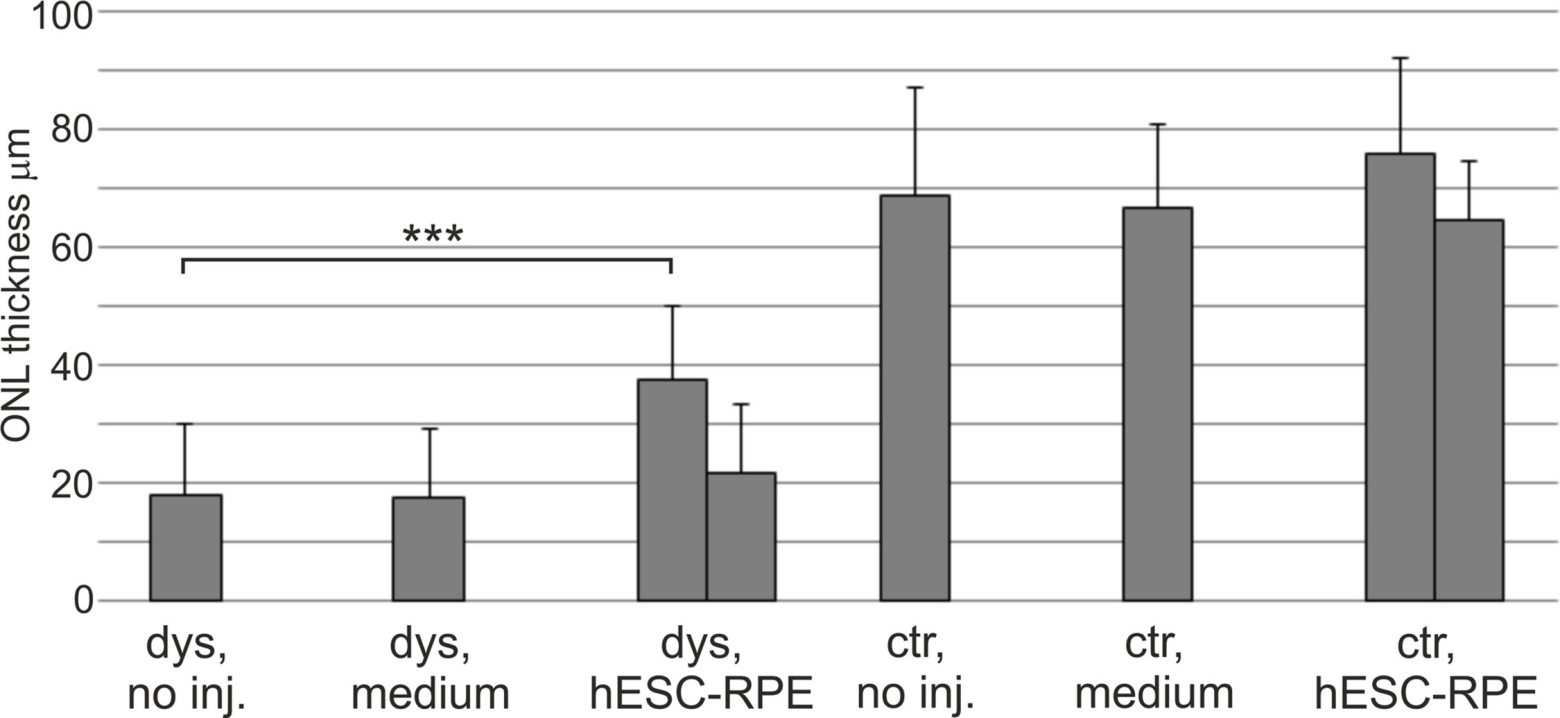
ONL thickness following subretinal injections in dystrophic and non-dystrophic RCS rats. ONL thicknesses were measured from HE samples of rats euthanized 41 days post-injection. Each column represents one rat eye. Measurements were performed with ImageJ software. Error bars show standard deviation.

**Fig 3:**
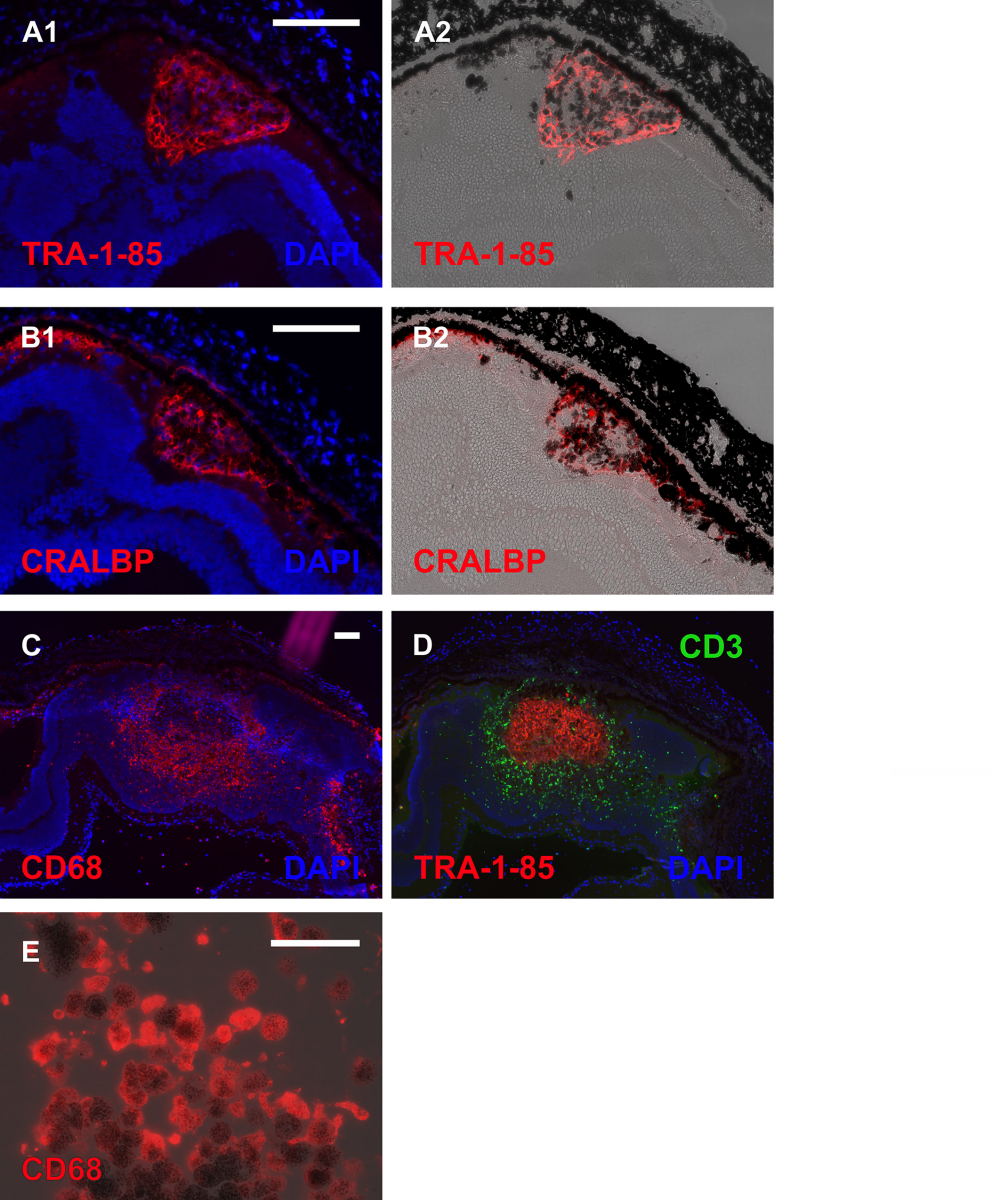
Implanted eyes of dystrophic RCS rats. Immunohistochemical staining showing labeling of injected cells 41 days post-injection with antibodies against human antigen TRA-1-85 (A1-2), RPE marker CRALBP (B1-2, antibody recognizes also rat CRALBP), macrophage marker CD68 (C), and T-cell marker CD3 (D). Large pigmented cells detected 105 days post-injection were positive for CD68 antibody (E). Scale bar 100 μm in A-D and 50 μm in E.

At day 37 post-injection, some functional rescue of ERG signal was detected in hESC-RPE injected dystrophic eyes (Fig 1) and in one animal ONL thickness was also significantly (p < 0.001) preserved (Fig 2). Slight functional rescue (Fig 1) but no ONL preservation (Fig 2) was detected in the dystrophic eye injected with medium only. In non-dystrophic rats, the ERG response was slightly lowered in hESC-RPE injected eyes and slightly increased in medium injected eyes compared to non-injected parallel eyes (Fig 1), but no significant changes were observed in the ONL thickness (Fig 2). Human antigen TRA-1-85 staining showed that 41 days post-injection most of the injected cells were located subretinally in different sized cell clusters in all rats (Fig 3A). Injected hESC-RPE cells also remained positive for RPE marker CRALBP (Fig 3B). In dystrophic eyes, infiltration of CD68 positive cells was observed in the outer segment debris zone, also without injection (data not shown), and around the injected hESC-RPE cells (Fig 3C). In one of the dystrophic eyes, infiltration of CD3 positive cells was also present around the injected cells (Fig 3D). In non-dystrophic eyes, CD68 positive cells were detected in the subretinal space only in the hESC-RPE injected eyes, mainly around the injected cells and no CD3 positive cells were detected (data not shown). Clusters of large pigmented cells were present even after 105 days post-injection. However, these cells were not positive for TRA-1-85 or CRALBP. Instead, the pigmentation appeared to co-localize with CD68 staining (Fig 3E). No intraocular tumors were detected.

### Surface and permeability characterization of ultrathin PI carrier

**Fig 4:**
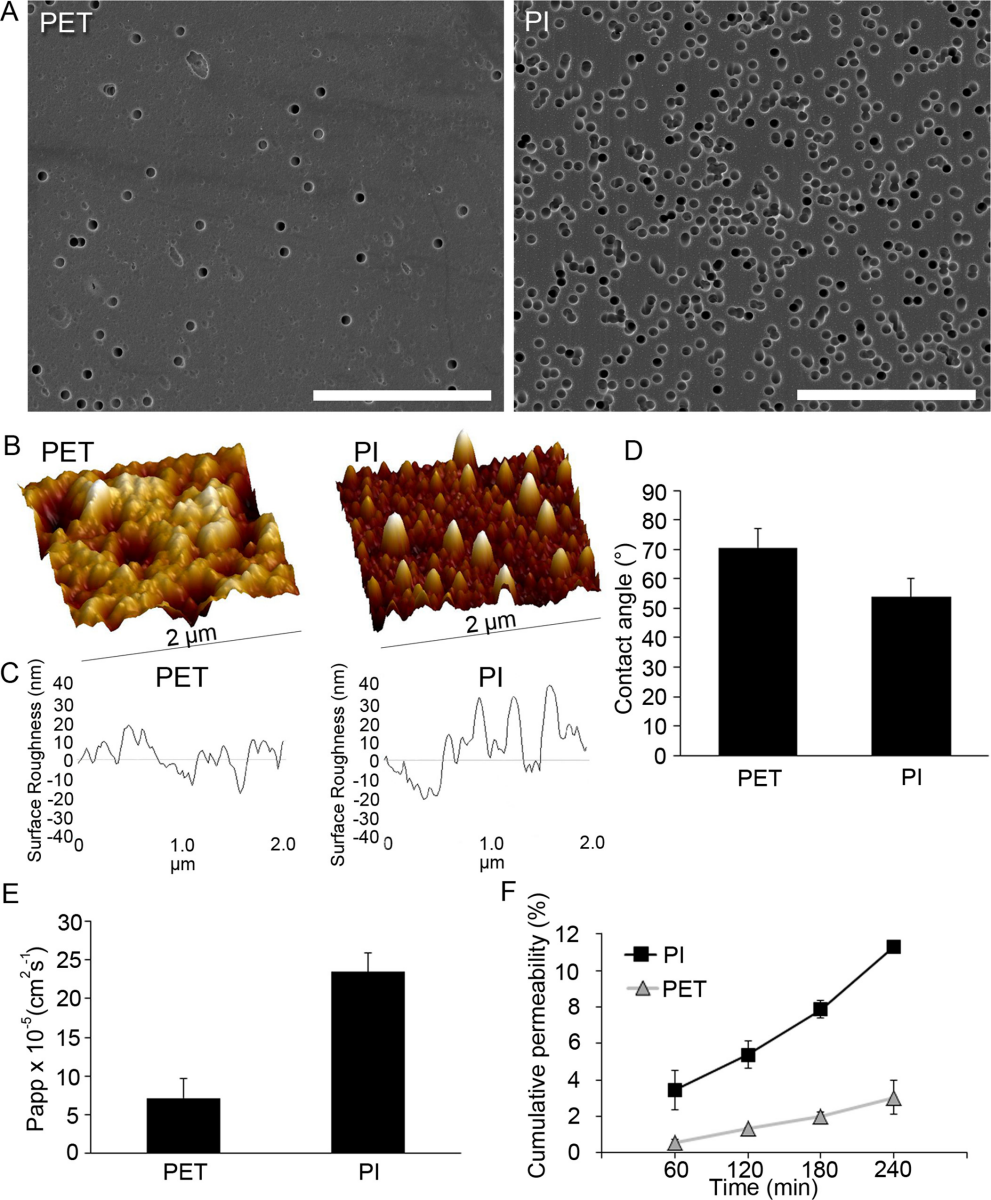
PI and PET membrane surface and permeability characteristics. SEM micrographs of PET and PI surface topography and pore distribution, scale bars 20 μm (A). AFM 3D images (B) and line profiles (C) demonstrating the surface roughness of PET and PI. Contact angle measurements of PET and PI (D). The apparent permeability coefficient Papp (E) and the cumulative permeability of small molecular weight fluorescent marker (F) for the PET and PI.

For subretinal sheet transplantation of hESC-RPE cells, the suitability of ultrathin PI membrane was examined. SEM was used to evaluate the average pore distribution of PI. PET membrane, a commonly used RPE culture substrate [18, 28, 30, 31], was analyzed for comparison. SEM showed that PI membranes had higher pore density compared to PET (Fig 4A). AFM revealed a clear nanotopography on both membranes (Fig 4B and 4C). PI had slightly rougher surface morphology (Rq 26.2±9.7 nm) compared to PET (Rq 13.5±3.8 nm). Contact angle measurements (Fig 4D) demonstrated slightly lower values in the surface wettability for PI (average 53.9±6.0°) compared to PET (average 70.6±6.4°). PI membranes also demonstrate higher Papp-value (2.34 x 10–4± 2.47 x 10–5 cm^2^s^-1^) compared to the Papp for PET (7.15 x 10–5±2.57 x 10–5 cm^2^s^-1^, Fig 4E) and larger increase in cumulative permeability of the small molecular weight substance over time. After four hours, PI membranes showed high cumulative permeability of 11.3±0.3%, whereas notably lower cumulative permeability of 3.0x0.9% was recorded for the PET membranes (Fig 4F).

### PI membrane transplantation of hESC-RPE into rabbit eye

**Fig 5:**
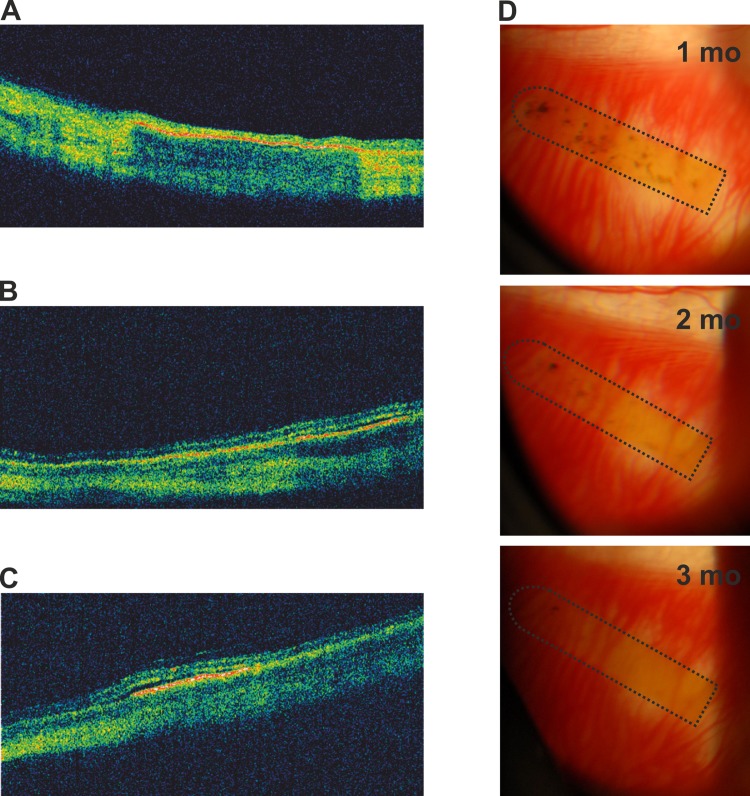
In vivo follow-up of hESC-RPE-PI implanted rabbit eyes. OCT scans showing hESC-RPE-PI in three different rabbit eyes three months after transplantation (A-C). Representative fundus photographs showing hESC-RPE-PI (marked with dotted line) of one rabbit one, two and three months after transplantation (D).

Apart from cataract formation in some of the rabbits, there were no other significant surgical complications. OCT showed good placement of the membranes (Fig 5A–5C). In one rabbit with hESC-RPE-PI some subretinal fluid on the membrane was detected (Fig 5C). A gradual loss of pigmentation on hESC-RPE-PI was observed over time (Fig 5D). Over two months, a-wave ERG responses remained roughly equal in eyes with hESC-RPE-PI or PI alone compared to the fellow eyes. In one rabbit with hESC-RPE-PI, the a-wave amplitude was clearly reduced in the operated eye (S2 Fig). On the other hand, after two months the b-wave amplitudes were clearly reduced in all eyes, operated and non-operated, compared to the amplitudes measured prior surgery (S2 Fig). At the three-month end point, ERG measurements were discarded due to cataract formation.

**Fig 6:**
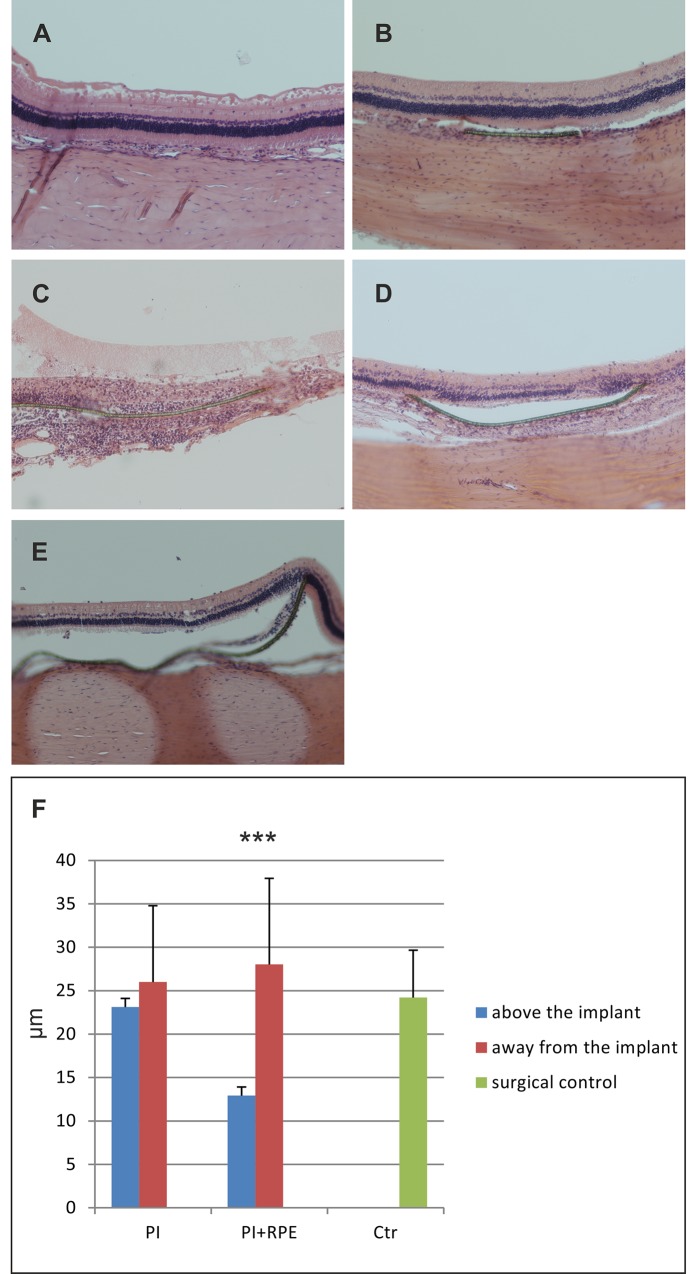
Histological analyses of rabbit retinas three months post-transplantation. HE staining of surgical control (A), PI alone (B) and hESC-RPE-PI (C and D). ONL thicknesses measured from HE samples, surgical control (n = 1), PI alone (n = 3), hESC-PI (n = 5) (E). Measurements were performed with ImageJ software. Error bars show standard deviation.

Three months after transplantation, histological analysis showed no evidence of intraocular tumors. In the surgical control or eyes with PI alone, no obvious signs of inflammation or significant retinal atrophy were observed (Fig 6A and 6B). However, in eyes transplanted with hESC-RPE-PI, mononuclear cell infiltration was observed around two out of five membranes and the ONL was totally destroyed (Fig 6C). In two out of five hESC-RPE-PI transplanted eyes, ONL was disorganized and the number of nuclei was reduced either over whole or part of the membrane (Fig 6D). In one eye with hESC-RPE-PI, the ONL thickness was only slightly reduced and ONL nuclear density was somewhat preserved compared to the areas away from the membrane (Fig 6E). Overall, ONL thickness was significantly (p < 0.001) reduced over hESC-RPE-PI compared to regions away from the membrane (Fig 6F).

The hESC-RPE-PI transplantations were performed with surgical forceps. In addition to difficulties in controlling the membrane during delivery, there is a high risk for damaging the hESC-RPE cells. Thus, we subsequently studied the use of a metallic shooter instrument with plain ultrathin PI. Despite multiple intraoperative attempts, delivery of the ultrathin PI proved incompatible with the shooter instrument in its current form as the membrane was too pliable, folded around the edges of the instrument during ejection or would be bend at the retinotomy.

## Discussion

Differentiation of RPE cells from hPSCs have made cell therapy a potential treatment for RPE-related diseases [4, 32]. Recent clinical trials with hESC-RPE cell suspensions injected subretinally have so far reported no major adverse effects related to the transplanted cells [7, 8, 33]. However, for successful treatment, the suspension transplanted cells need to adhere on the Bruch’s membrane and be integrated into the existing RPE layer to escape cell death and potential immune rejection and to function properly. In the pathologic environment of diseased retina, reaching sufficient level of integration may prove to be a difficult task. It is known that RPE attaches poorly to diseased or aged Bruch’s membrane [34–36]. In animal models, unattached cells have formed large cell clusters and the nonintegrated cells have been eventually cleared, potentially by macrophages [13, 37, 38]. Likewise, in our cell suspension experiments, the injected hESC-RPE cells were mainly detected in cell clusters and after 105 days no TRA-1-85 positive cells could be identified. Instead, the pigmented cells present stained with anti-CD68 being potentially macrophages which had engulfed the transplanted cells. Short-term, the hESC-RPE cells survived in the subretinal space and some functional rescue of ERG signals in the hESC-RPE injected dystrophic rat eyes was detected. Curiously, minor functional rescue/improvement of ERG signal was detected also in medium injected eyes in dystrophic and non-dystrophic rats. It is possible that e.g. macrophages potentially recruited to the injection site cause this effect by phagocytosis and/or secretion of trophic factors, thus promoting survival or function of the photoreceptors. Indeed, other cell types than RPE, such as mesenchymal stem cells, have shown similar protective effects following subretinal injection into RCS rats. The underlying mechanisms still remain mainly elusive, but recent data suggests that activation of endogenous regenerative mechanisms such as the progenitor potential of retinal Müller cells may play a role, in particular since a therapeutic benefit does not require the cells to persist in the transplantation site [39–42].

In order to increase the survival rate of the transplanted cells and to reconstruct or replace the damaged Bruch’s membrane, efforts have been made to develop a suitable RPE-scaffold implant [13, 14, 16–20, 43]. The requirements for the scaffold include support of RPE monolayer growth and functionality, bio- and immune compatibility, and surgical manageability. Carrier porosity/permeability is important, especially if the scaffold is not rapidly biodegradable. Elastic modulus is another important parameter, not only to avoid problems like retinal detachment but to support proper RPE function like phagocytosis which has been shown to be decreased on firm substrates compared to more flexible ones [44]. To date, there have been no published large animal studies with transplantation of hPSC-derived RPE on a supportive scaffold. We have previously shown that PI is a suitable culture surface for hESC-RPE [25]. In the current study, we further examined the porosity and permeability of ultrathin PI and its suitability for subretinal transplantation. The pore density of the studied PI membranes was good and the pores were evenly distributed. In culture, no dome formation, a typical feature of transporting epithelia on non-permeable surfaces [45, 46], was detected on the PI membranes. PI was also well tolerated in the subretinal space. Since the examined PI membrane was very thin and flexible, it conformed well to the shape of the surrounding space and post-surgical retinal detachments were not observed. Due to the merangiotic nature of rabbit retina, most of the blood supply of the inner retina is derived from the choriocapillaris [47], which would likely lead to rapid retinal damage, if blocked by a non-permeable implant. In a previous study by Montezuma et al. 2006 [24], the authors reported disorganization of the ONL when non-perforated PI implants were transplanted in pigs, despite the fact that pigs have both choroidal and retinal arterial blood supply to the retina. In our study, the retinal nuclear layers were well preserved over the PI even three months post-transplantation, indicating good permeability and biocompatibility. On the other hand, when hESC-RPE-PI were transplanted, varying level of disorganization of the ONL and loss of the photoreceptors were detected in most of the rabbits. In two out of five rabbits, there was mononuclear cell infiltration around the hESC-RPE-PI. We also noticed a gradual loss of pigmentation on the membranes, most likely due to the loss of hESC-RPE and not depigmentation since no TRA-1-85 positive cells were detected on the implants. Graft rejection in allogeneic and xenogeneic transplantations is a major obstacle. Although eye is considered as an immune privileged organ and RPE as immunomodulatory tissue, subretinal transplantation experiments with both allografts and xenografts in several animal models have demonstrated that the immunological privilege of the subretinal space is imperfect or may be easily compromised [13, 34, 37, 48, 49]. Recent studies indicate, that transplanted allogeneic iPSC-RPE cells induce an innate immune response which cannot be overcome by the immunomodulatory mechanisms in the eye [11, 49]. One major factor in ocular immune privilege is the blood-retina barrier [50]. This barrier may be broken by diseases, such as AMD, or surgical procedure, exposing the transplanted tissue to immune rejection. Although hPSCs express low levels of MHC-I and β2-microglobin at their surface, expression of these proteins increases upon RPE differentiation. The cells may also acquire immunogenic features during prolonged culture, especially if exposed to xeno-products and undefined factors, such as serum [51–53]. Additionally, exposure of iPSC-RPE cells to proinflammatory cytokine interferon-gamma increases their MHC-II expression. These factors can predispose allogeneic implants to immune rejection of [11]. In our study, immunosuppressant was administered in the drinking water. This may be problematic, because water consumption of the animals varied substantially (daily intake of each animal was between 3%-100% of total water volume) leading potentially to variation in the cyclosporine plasma levels. As we did not examine this, insufficient immunosuppression could at least partly explain the loss of transplanted cells. The type and dose of immunosuppressant, administration route, and duration of immunosuppression remain to be studied further. It may also be, that by using autologous iPSC-RPE cells no immunosuppression is needed, as these cells seem to be immune tolerated [11, 54]. For future therapeutical use, ability to reduce the immunogenicity of allogeneic cultured RPE cells, or use of autologous or HLA-homozygous hiPSCs for better immunological compatibility, would greatly facilitate the application of hPSC-based RPE cells therapies.

Surgical manageability is an important factor contributing to the success of transplantation. In the study by Diniz *et al*. 2013, hESC-RPE were transplanted as a sheets on ultrathin parylene membranes into the subretinal space of rats. Many of the complications detected in their study, including damage to the transplanted RPE and increased cell reaction at the implant area, could potentially be explained by the surgical technique involving choroidal incision commonly used in animals with small eyes and large crystalline lenses [13]. In our study rabbit was chosen as the model animal due to its larger eye size allowing the use of surgical techniques similar to clinical procedures. However, although the biocompatibility of the ultrathin PI was good, the surgical technique still requires further development. During the hESC-RPE-PI transplantations, the membrane was exposed, potentially leading to damage to the hESC-RPE. To protect the cells, we tested the use of a shooter instrument with ultrathin PI. However, in their present forms, the ultrathin PI and the shooter instrument were not compatible as PI was too flexible. Flexibility is a typical problem also with other ultrathin substrates, such as parylene [20]. Further modifications to improve the surgical manageability of the ultrathin PI membrane would facilitate its usefulness in the future. Such enforcement could e.g. be added as a transient feature, by encapsulating the implant into rapidly biodegradable hydrogel. Alternatively delivery strategies may comprise injection through BSS or viscoelastic, in analogy to the approach by the Japanese hiPSC-RPE clinical trial [11].

Safety of transplanting cells differentiated from PSCs remains an important aspect. Although so far in the animal and human trials no major concerns have arisen, the safety of hPSC-derived cells in therapeutical use awaits final confirmation. In our study, the transplanted cells did no longer express pluripotency markers *OCT4* and *NANOG*. Additionally, no major adverse effects such as tumor formation were detected either in the rat or rabbit eyes.

In summary, ultrathin, porous PI was well tolerated in the subretinal space and preservation of ONL was observed even after three months of transplantation. We believe, that with proper modifications and protection of the RPE during transplantation, ultrathin PI is a promising scaffold material for therapeutic subretinal transplantation of RPE cells.

## Supporting Information

S1 Fig Gene and protein expression profiles of hESC-RPE prior injection into rats. RT–PCR analysis of eye/RPE and pluripotency marker genes, genomic control reactions excluding the enzyme are marked ‘-RT’ (A). Cytospin preparations showing the immunocytochemical staining of RPE/eye markers bestrophin-1, MITF, CRALBP, and PAX6, scale bar 50 μm (B). Live/Dead cell viability assay showing live cells stained with Calcein-AM (green) and dead cells with EthD-1 (red), scale bar 100 μm (C).
(TIF)

S2 Fig Dark-adapted ERG responses following PI transplantation into rabbit eyes. Fig shows averaged (two responses) flash ERGs recorded at 25 000 mcds/m^2^. Responses measured prior operation are in black and responses two months after transplantation are in red.
(TIF)

S1 Materials and Methods (DOCX)

S1 Table Primary antibody information. (DOCX)

S2 Table RT-PCR Primer sequences. (DOCX)
