# Supplementary figures and images for: Ultrathin Polyimide Membrane as Cell Carrier for Subretinal Transplantation of Human Embryonic Stem Cell Derived Retinal Pigment Epithelium
Source: PLoS One. 2015 Nov 25;10(11):e0143669. doi: 10.1371/journal.pone.0143669 (PMC4659637; doi:10.1371/journal.pone.0143669)

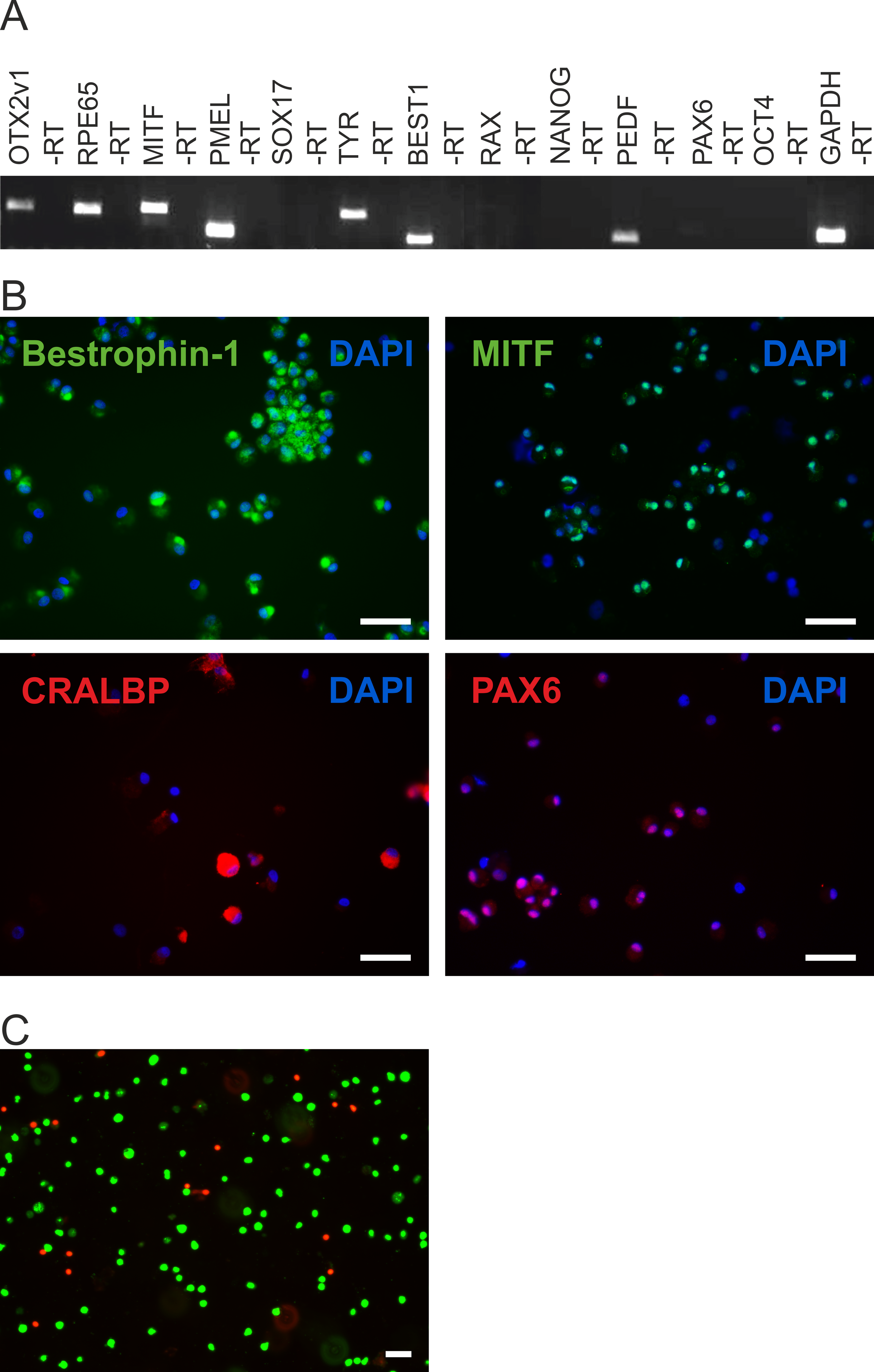

Supplement: S1 Fig — RT–PCR analysis of eye/RPE and pluripotency marker genes, genomic control reactions excluding the enzyme are marked ‘-RT’ (A). Cytospin preparations showing the immunocytochemical staining of RPE/eye markers bestrophin-1, MITF, CRALBP, and PAX6, scale bar 50 μm (B). Live/Dead cell viability assay showing live cells stained with Calcein-AM (green) and dead cells with EthD-1 (red), scale bar 100 μm (C). (TIF) [file pone.0143669.s001.tif]

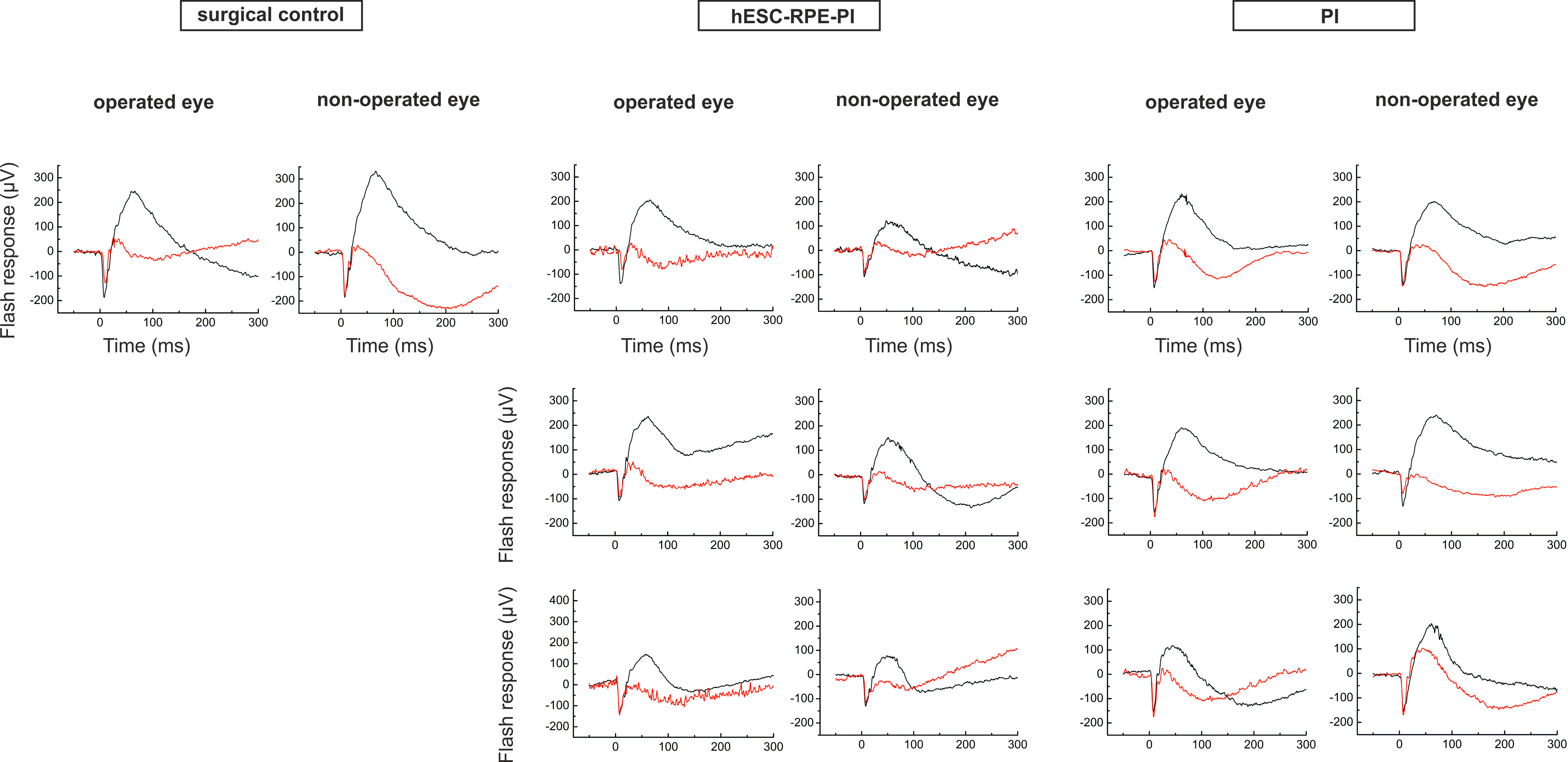

Supplement: S2 Fig — Fig shows averaged (two responses) flash ERGs recorded at 25 000 mcds/m2. Responses measured prior operation are in black and responses two months after transplantation are in red. (TIF) [file pone.0143669.s002.tif]
