# Supplementary material for: Ultrathin Polyimide Membrane as Cell Carrier for Subretinal Transplantation of Human Embryonic Stem Cell Derived Retinal Pigment Epithelium
Source: PLoS One. 2015 Nov 25;10(11):e0143669. doi: 10.1371/journal.pone.0143669 (PMC4659637; doi:10.1371/journal.pone.0143669)
Supplement: S1 Materials and Methods — (DOCX) [file pone.0143669.s003.docx]

# S1 Materials and Methods

## Transplantation – Cell suspension injection studies in rats

## RT-PCR

Prior hESC-RPE injection, gene expression of specific RPE/eye-related genes *OTX2v1*, *RPE65*, *MITF*, *PMEL*, *SOX17*, *TYR*, *BEST1*, *RAX*, *PEDF* and *PAX6* as well as pluripotency markers *NANOG* and *OCT4* was assessed with RT-PCR. GAPDH was used as an endogenous control. Total RNA was extracted using NucleoSpin RNA II kit (Macherey-Nagel, GmbH & Co, Düren, Germany) according to the manufacturer's instructions. Genomic control reactions without reverse transcriptase were prepared for each sample. Detailed RT-PCR protocol has been previously reported [26]. The specific primer sequences are listed in Supporting Table S5.

## Immunocytochemistry

The expression of PAX6, MITF, bestrophin-1, and CRALBP in the injected hESC-RPE cells was assessed with immunocytochemistry. For the analysis, cells were spun onto object glasses using the Cellspin II cytocentrifuge (Tharmac, GmbH, Waldsolms, Germany) 600 rpm for 5 min. After spinning, the cells were fixed with 4 % paraformaldehyde for 20 min RT. Immunocytochemical staining was performed as previously described [26]. Samples were incubated with the following primary and secondary antibody dilutions: anti-MITF 1:350, anti-bestrophin 1:500, anti-CRALBP 1:1000 and anti-PAX6 1:200, Alexa Fluor 568-conjugated goat or donkey anti-mouse IgG, and Alexa Fluor 488-conjugated donkey anti-rabbit IgG (all secondaries 1:800 and from Molecular Probes, Life Technologies). Primary antibody information is listed in Supporting Table S2. Images were captured with Olympus IX51 fluorescence microscope (Olympus).

The viability of injected cells kept in suspension for 6 h at +4 °C was analyzed with LIVE/DEAD® Cell Viability Assay kit (Life Technologies) according to manufacturer’s instructions. The labeled cells were imaged with Olympus IX51 fluorescence microscope.

## Human ESC-RPE cell suspension injection into rat eyes

One day before harvesting, 10 mM ROCK-specific inhibitor Y-27632 (Tocris, Bristol, UK) was added to the cells. Cells were harvested with 1x Trypsin-EDTA. After neutralizing with human serum (GE Healthcare, Buckinghamshire, UK), the cells were filtered with 40 µm cell strainer and resuspended in RPEbasic medium to a final concentration of 50 000 cells/µl. Cells were kept on ice until injected.

Only the right eye was injected in each rat. Rats underwent surgery at postnatal days 22-29. Rats were anesthetized by abdominal injection of medetomidine (0.4 mg/kg) and ketamine (60 mg/kg). Additionally, topical anesthesia was administered with Oxybuprocaine hydrochloride solution (Santen). Pupils were dilated with tropicamid (Santen). Two microliters of cell suspension (containing about 100 000 cells) or medium alone was injected subretinally into two separate locations per eye. Injections were performed transclerally using a 30 gauge needle attached to a Hamilton syringe with the aid of viscotears and a glass coverslip. After surgery, anaesthesia was counteracted with 1 mg/kg atipamezole (Zoetis, NJ, USA). Rats received 5 mg/kg cyclosporin A (Novartis, Basel, Switzerland) in drinking water starting two days before the transplantation until the end of follow-up and as daily subcutaneous injections for three days post-transplantation.
