# Supplementary material for: Ultrathin Polyimide Membrane as Cell Carrier for Subretinal Transplantation of Human Embryonic Stem Cell Derived Retinal Pigment Epithelium
Source: PLoS One. 2015 Nov 25;10(11):e0143669. doi: 10.1371/journal.pone.0143669 (PMC4659637; doi:10.1371/journal.pone.0143669)
Supplement: S1 Table — (DOCX) [file pone.0143669.s004.docx]

**S1 Table. Primary antibody information.**

| ***Name*** | ***Host and clonality*** | ***Supplier and catalog reference*** |
| --- | --- | --- |
| Anti-CRALBP | Mouse monoclonal | Abcam Cambridge, UK; ab15051 |
| Anti-CD68 | Mouse monoclonal | Serotec, Kidlington, UK; MCA341GA |
| Anti-CD3 | Goat polyclonal | Santa Cruz Biotechnology, TX, USA; sc-1127 |
| Anti-TRA-1-85 | Mouse monoclonal | Peter Andrews, University of Sheffield, UK |
| Anti-MITF | Rabbit polyclonal | Abcam Cambridge, UK; ab 59232 |
| Anti-bestrophin | Rabbit polyclonal | Abcam Cambridge, UK; ab14928 |
| Anti-PAX6 | Mouse monoclonal | DSHB Hybridoma Product, deposited by Kawakami, Atsushi |
