# Supplementary material for: Ultrathin Polyimide Membrane as Cell Carrier for Subretinal Transplantation of Human Embryonic Stem Cell Derived Retinal Pigment Epithelium
Source: PLoS One. 2015 Nov 25;10(11):e0143669. doi: 10.1371/journal.pone.0143669 (PMC4659637; doi:10.1371/journal.pone.0143669)
Supplement: S2 Table — (DOCX) [file pone.0143669.s005.docx]

**S2 Table. RT-PCR primer sequences.**

| ***Gene*** | ***forward primer sequence 5' > 3'*** | ***reverse primer sequence 5' > 3'*** |
| --- | --- | --- |
| *OTX2v1* | GGGCCCTGGGCTTCTTGTCC | ATTGGCCACTTGTTCCACTC |
| *RPE65* | TCCCCAATACAACTGCCACT | CACCACCACACTCAGAACTA |
| *MITF* | AAGTCCTGAGCTTGCCATGT | GGCAGACCTTGGTTTCCATA |
| *PMEL17* | GTGGTCAGCACCCAGCTTAT | GAGGAGGGGGCTATTCTCAC |
| *SOX17* | CGCACGGAATTTGAACAGTA | CACACGTCAGGATAGTTGCAG |
| *TYR* | TGCCAACGATCCTATCTTCC | GACACAGCAAGCTCACAAGC |
| *BEST* | GAATTTGCAGGTGTCCCTGT | ATCCTCCTCGTCCTCCTGAT |
| *RAX* | CTGAAAGCCAAGGAGCACATC | CTCCTGGGAATGGCCAAGTTT |
| *NANOG* | TGCAAATGTCTTCTGCTGAGAT | GTTCAGGATGTTGGAGAGTTC |
| *PEDF* | AGCTCGCCAGGTCCACAAAG | TGGGCAATCTTGCAGCTGAG |
| *PAX6* | AACAGACACAGCCCTCACAAACA | CGGGAACTTGAACTGGAACTGAC |
| *OCT4* | CGTGAAGCTGGAGAAGGAGAAGCTG | AAGGGCCGCAGCTTACACATGTTC |
| *GAPDH* | GTTCGACAGTCAGCCGCATC | GGAATTTGCCATGGGTGGA |
